# An evaluation of a virtual musculoskeletal podiatry service implemented to address prolonged National Health Service waiting times

**DOI:** 10.1002/jfa2.12039

**Published:** 2024-10-18

**Authors:** Agalliu Brunilda, Tunprasert Thanaporn, Walton Tom, McManus Liam

**Affiliations:** ^1^ South West Podiatry London UK; ^2^ School of Sport and Health Sciences University of Brighton Eastbourne UK

**Keywords:** musculoskeletal, podiatry, virtual

## Abstract

**Background:**

The COVID‐19 pandemic had a substantial impact on healthcare systems globally, particularly in the public sector. To address the challenges posed by the pandemic, musculoskeletal (MSK) healthcare providers had to rapidly adopt virtual platforms for delivering care, representing a major shift in how healthcare was delivered.

**Objective:**

This manuscript aims to retrospectively evaluate a virtual MSK podiatry service offered by a private provider under a National Health Service commission, in terms of patient access, waiting times and patient‐reported pain. This service was developed and implemented in response to the COVID‐19 pandemic and the extended waiting times.

**Methods:**

A retrospective clinical service evaluation was conducted on MSK podiatry services delivered via telephone or virtual consultations. The evaluation covered a cohort of 574 referred patients over a 19‐month period (July 2021 to January 2023). It analysed demographic data, initial and final visual analogue pain scores, pathology categories, orthoses prescriptions and exercise rehabilitation plans.

**Results:**

Data from a total of 492 patients (male = 152 and female = 340) were analysed, with 82 patients excluded for non‐attendance. The average waiting time from referral‐to‐first appointment and referral‐to‐discharge was 35 and 91 days, respectively. Results showed statistically significant improvement (*p* < 0.001) in the mean visual analogue scale when patients received orthoses with and without a rehabilitation plan (4.12 ± 2.55 and 3.33 ± 2.88, respectively). Most patients (61.5%) were aged 40–69, with “foot pain” being the main reported pathology category. Patients had an average of two appointments. 56.5% of patients remained virtual throughout their journey and were successfully discharged to self‐management. 43.9% were discharged to other face‐to face services.

**Conclusions:**

The study provided evidence that the virtual MSK podiatry service achieved a statistically significant reduction in patient‐reported pain for various pathologies with reasonable waiting times. The service delivered favourable outcomes and complemented traditional services at a time with limited access due to the COVID‐19 pandemic.

**Abbreviations**
AHP: allied health professions
HCPC: the health and care professions council
MSK: musculoskeletal
NHS: National Health Service
PMS: practice management software
SWP: South West Podiatry
VAS: visual analogue scale

## 1 INTRODUCTION

In recent decades, substantial technological developments have created an opportunity to provide healthcare services through virtual platforms. The concept of digital healthcare holds significant promise, as it offers the potential for cost‐effective and accessible medical care that transcends geographical boundaries and time constraints. The widespread use of internet‐connected devices has played a pivotal role in enabling this approach to healthcare provision. Moreover, virtual platforms can enable efficient patient triage, assessment and timely intervention [1]. These approaches are revolutionising the delivery of healthcare services, offering new opportunities for the provision of accessible, efficient and patient‐centred care [2, 3]. While telehealth offers numerous advantages, it is important to acknowledge that diagnosing some conditions can be more challenging remotely compared to in‐person consultations due to the lack of a physical examination. However, technological advancements and assessment tools are continuously improving the effectiveness of remote consultations.

The COVID‐19 pandemic has drastically impacted global healthcare, particularly in the public sector, including the UK's National Health Service (NHS). The NHS had to adapt its caseload and prioritise overwhelmed emergency and acute services, resulting in a substantial backlog of patients on primary, secondary and tertiary care waiting lists [4]. Musculoskeletal (MSK) departments have encountered notable challenges as MSK conditions, which represent up to 21% of the annual caseload within primary care in England, require considerable resources and often involve a physical examination for diagnosis [5]. To address these challenges, virtual platforms played a crucial role in delivering remote healthcare services [6]. Virtual services offer a solution for MSK patients who are unable to attend in‐person appointments, providing a convenient means of consultation, diagnosis and management. Furthermore, virtual consultations have shown potential for reducing healthcare costs and waiting times associated with traditional face‐to‐face services. They are also considered an eco‐friendly and sustainable alternative [7, 8]. This makes virtual services a promising option, not only for rural areas but also for urban environments with limited transportation [9]. However, there are concerns regarding their efficacy and more studies are required reporting patient outcomes.

During the COVID‐19 pandemic, the Royal College of Podiatry published a commentary on virtual podiatry services, mainly focussing on triaging patients based on the level of viral exposure [10]. However, no further United Kingdom podiatry‐specific guidelines on virtual services have been published to date. This was also highlighted in a recent scoping review of the literature which called for additional research studies reporting the outcomes of virtual podiatry services [11]. This manuscript aims to present the results of a service evaluation of an NHS commissioned virtual MSK service delivered in the private sector. The evaluation focused on the effectiveness of treatments offered in improving symptoms and assessing the management of patients. This is one of few studies conducted to investigate the impact of virtual MSK podiatry services during the COVID‐19 pandemic on waiting times and outcomes, providing valuable insights into their effectiveness.

## 2 METHODS

### 2.1 Design

This study is a retrospective evaluation of the virtual MSK services provided to NHS patients by a private podiatry clinic. After consultation with the NHS Research and Development team, it was determined that this investigation did not require an ethics approval from the NHS Research Ethics Committee. Additionally, consent and approval were obtained from both the NHS and private data controllers.

### 2.2 Settings and participants

South West Podiatry (SWP) is an independent private practice based in London, UK. In July 2021, SWP was commissioned to offer a virtual MSK podiatry service to NHS patients across two boroughs (NHS Greenwich CCG and NHS North Hampshire CCG). The combined population of the two boroughs is approximately 420.000 with diverse socioeconomic backgrounds living in both urban and rural areas [12].

### 2.3 Virtual podiatry service protocols

**FIGURE 1:**
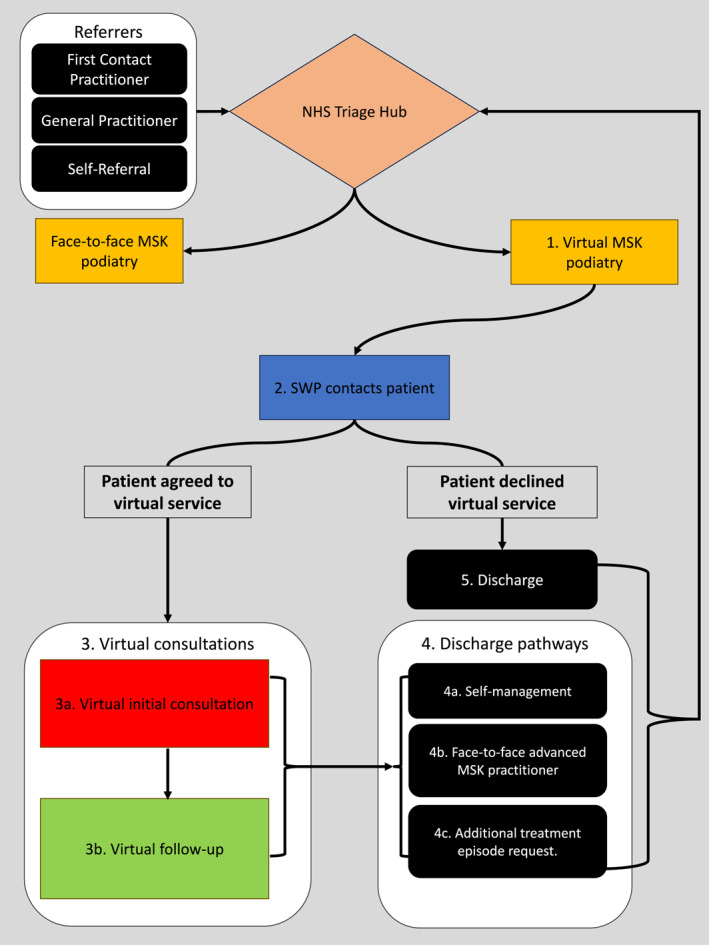
Flowchart showing major steps of the clinical pathway. Various referral sources reached the NHS Triaging Hub which after evaluation were assigned to either the face‐to‐face service or the virtual service (1). Patients were contacted by SWP (2) to determine if they can be admitted to the virtual consultation pathway (3). Discharge pathways following consultations (4) and without consultation (5) are also shown. NHS, National Health Service; SWP, South West Podiatry.

In response to the demands created by the pandemic, SWP developed a virtual podiatry service for private patients, offering both video and telephone consultations. This includes a development of an internal best practice guidance. Our guidelines were compiled from sources outside of podiatry and considered; the health and wellbeing of the practitioner and the patient, the patient's choice to accept or reject virtual, data protection, reviewing the patient's environment, dynamic risk assessments, what constitutes an adequate clinical examination, patient reported outcome measures and record keeping standards in‐line with the Health and Care Professions Council (HCPC) guidelines [13, 14, 15, 16]. The service consisted of an initial virtual consultation and up to two follow‐up appointments. Major steps of the patient pathway are shown in a flowchart (Figure 1).

The NHS triage hub assessed each referral and determined the patient's suitability and pathway. Referral into MSK podiatry consisted of a choice between face‐to‐face podiatry and virtual services (Figure 1–step 1). As a result of significant waiting lists, patient wishes were not factored into this decision‐making process. Patients were then sent an invitation letter by SWP to schedule their initial virtual consultation within 14‐days (Figure 1–step 2). If no response was received, this was followed‐up with a telephone call. Patients who declined to enter the virtual service were discharged back to the NHS Triage Hub (Figure 1–step 5). Patients agreeing to the virtual service were scheduled for an initial virtual consultation (Figure 1–step 3), providing access to a HCPC registered podiatrist for advice, assessment and management offered by SWP. After a maximum of three virtual consultation sessions, patients were discharge to either: (a) self‐management, (b) a face‐to‐face advanced MSK practitioner or (c) back to the NHS Triaging Hub requesting an additional treatment (Figure 1–step 4).

The virtual consultations comprised of weight‐bearing observations and assessments of foot posture index, dynamic range of motion, strength assessments of the foot, ankle and lower leg, foot deformities and alignment and examination of footwear [17, 18]. These mirrored the assessments conducted in an in‐person weight bearing consultation. Clinicians relied on patients' descriptions and guidance to pinpoint the exact anatomical location of their painful symptoms to provide a tailored treatment plan. Management included sending educational content via SMS, email or post. Podiatrists could also send patients rehabilitation via online software and design orthoses which were sent to the patient's home via tracked delivery. Patients used smartphones, tablets and PCs to access a link that was provided to them via e‐mail and SMS to initiate the virtual consultation. This information was also summarised in an email sent to them after the consultation. If required, an orthoses request prescription would be created by the podiatrist, sent to SWP's in‐house orthotic workshop where it was processed. Orthoses were prescribed based on the patient's presenting complaint, pathology category and symptomatology, with variations in the orthotic prescription depending on the anatomical location of the affected area. Prescribed orthoses were designed from prefabricated Slimflex or Interpod devices (Algeos Ltd, UK). To enhance consistency in our clinical, operational and governance practice, standard operating procedures were created detailing the service steps and including incident report form procedures, NHS orthoses prescription steps, virtual guidelines and written and video instruction manuals.

### 2.4 Data collection and processing

During the virtual consultations, patients were asked by the attending clinician to answer a series of questions following a structured form on the practice management software (PMS). Two clinicians (BA and LM) were responsible for extracting data from the PMS. Following data extraction, it was encrypted and anonymised restricting access to authorised personnel. Additional parameters were calculated using Microsoft Excel which included patient demographics (such as age and gender), referral date, first appointment date, discharge date, number of appointments, pathology category, initial assessment, initial and discharge visual analogue scale (VAS) scores (0‐‘no pain’ to 10‐‘worst pain imaginable’), information on orthoses prescription, exercise rehabilitation plan and discharge summary action. This process ensured compliance with the clinic's data protection policies as well as the General Data Protection Regulation guidelines and Data Protection Act 2018 [19].

### 2.5 Data analysis

The data were analysed using the SPSS Software (version 28.0.1.1) by TT. Descriptive statistics, such as mean, standard deviation, minimum and maximum, were used to evaluate the quality of the MSK virtual service provided to patients. The related‐samples Wilcoxon signed‐rank test was employed to compare differences between initial and discharge VAS scores, with significance set at *p* < 0.05. Sub‐group analyses were performed based on age, gender, pathology category and discharge summary action.

## 3 RESULTS

### 3.1 Patient demographics, attendance and waiting times

From July 2021 to January 2023, a total of 574 patients were referred to the virtual services (Table 1). Most patients were female (*n* = 386, 67.3%). The most prevalent age groups were between 40 and 49 (*n* = 104, 18.1%), 50 and 59 (*n* = 138, 24.0%) and 60 and 69 (*n* = 111, 19.3%). Based on the referrals received, the most common pathology category was “foot pain” (*n* = 215, 37.5%). Most patients were seen for a total of 2 appointments (*n* = 253, 44.1%), whilst 82 (14.3%) opted not to engage with the virtual service. Thus, a total number of patients seen by the virtual service was 492.

**TABLE 1:** Patient demographics including gender, age, pathology category and number of appointments attended.

Categories	Patients (*n*)	Percentage (%)
*Total*	*574*	*100*
Gender		
Male	188	32.7
Female	386	67.2
Age group		
17–29	45	7.8
30–39	70	12.2
40–49	104	18.1
50–59	138	24.0
60–69	111	19.3
70–79	76	13.2
80–89	27	4.7
90–99	3	0.5
Pathology category		
Achilles tendinopathy	20	3.5
Flat foot	60	10.5
Foot pain	215	37.5
Hallux valgus	89	15.5
Metatarsalgia	7	1.2
Neuroma/bursa complex	46	8.0
Osteoarthritis	66	11.5
Plantar fasciitis	71	12.4

We were not able to establish a reason for 48 out of 82 patients who did not wish to proceed with a virtual consultation. For 34 patients, the reasons provided were: patient did not wish to use a virtual service (*n* = 24), patient was already under the care of other services (*n* = 4) and other reasons (e.g., an appointment was no longer required) (*n* = 6). No trend was found for a particular gender or age group.

For patients who chose to engage with the service (*n* = 492), the average waiting time until the first appointment was 34.6 days. SWP's contract agreement indicated a maximum waiting time of 18 weeks (126 days) from the initial referral date until the patient was seen. Most patients were seen within 3 months (*n* = 486, 98.8%). The average number of days between the initial referral and the discharge was 91.1 days. Patients accessed the virtual service for an average of 56.61 days prior to discharge. The standard deviations and range for each of the waiting times mentioned is shown in Table 2. The appointment number ranged from one to four appointments: one appointment (*n* = 99, 17.3%), two appointments (*n* = 253, 44.1%), three appointments (*n* = 134, 23.3%) and four appointments (*n* = 6, 1.1%). Patients were discharged either to self‐management (*n* = 276, 56.1%) or to face‐to‐face allied health professions services (*n* = 216, 43.9%). Patients in the former group (discharged to self‐management) had demonstrated a statistically significant reduction in pain (*p* < 0.0001), improved function or ability to self‐manage, requiring no further interventions.

**TABLE 2:** Virtual service waiting times between initial referral (Figure 1(2)), the first appointment (Figure 1(3.a)) and discharge (Figure 1(5, 4.a, 4b, or 4c)).

	Mean	Standard deviation	Min	Max
Day(s) between an initial referral and the first appointment	34.6	21.4	0	226
Day(s) between the first appointment and discharge	56.6	40.5	0	238
Day(s) between an initial referral and discharge	91.1	46.1	2	282

### 3.2 Patient management and outcomes

The management plans provided to patients during the initial consultation are summarised in Table 3. Most patients were prescribed with both orthoses and exercise rehabilitation plan (*n* = 277, 56.3%). Patients who received orthoses prescription showed a significant improvement in their VAS score, whether an exercise rehabilitation plan was included or not (4.1 ± 2.6, *p* < 0.0001 and 3.3 ± 2.9, *p* < 0.001, respectively). Conversely, patients who did not receive orthoses prescription, regardless of whether they had an exercise rehabilitation plan, showed little to no improvement in terms of their VAS score (0 and 0.1 ± 0.7, respectively).

**TABLE 3:** Management methods provided through the virtual service.

Categories	Patients (*n*)	Percentage (%)	VAS at initial appointment	VAS at discharge	VAS improvement (between an initial appointment and at discharge)	Significance
Orthoses and an exercise rehabilitation plan prescribed	277	56.3	6.9 ± 2.1	2.8 ± 2.6	4.1 ± 2.6	***p < 0*.*0001***
No orthoses but with an exercise rehabilitation plan prescribed	6	1.2	2.0 ± 2.2	2.0 ± 2.2	0 (no difference)	‐
Orthoses prescribed but with no exercise rehabilitation plan	123	25.0	6.6 ± 2.6	3.3 ± 3.5	3.3 ± 2.9	***p < 0*.*001***
No orthoses and no exercise rehabilitation plan	86	17.5	6.1 ± 3.9	6.0 ± 4.0	0.1 ± 0.7	***p = 0*.*18***

*Note*: Bold‐italic values indicate the significance.

The discharge outcome of patients with different pathology categories was evaluated and summarised in Table 4. For most categories, a similar number of patients were discharged to self‐management or other AHP care, with the exception of flat foot (*n* = 45, 16.3%) and plantar fasciitis (*n* = 50, 18.1%). These two pathologies showed satisfactory outcomes, resulting in a greater proportion of patients being discharged to self‐management. On the other hand, a substantial proportion of patients referred with foot pain (31.0%), hallux valgus (19.9%), osteoarthritis (14.4%) and neuroma/bursa complex (13.0%) required onward referral to other AHP services.

**TABLE 4:** Discharged groups by pathology category.

	Average VAS at initial appointment	Average VAS at discharge	Average VAS improvement	Discharged to self‐management	Discharged to AHP services
Achilles tendinopathy	7.2 ± 2.0	3.6 ± 2.8	3.6 ± 2.2	*n* = 10, 3.62%	*n* = 10, 4.63%
Flat foot	6.0 ± 2.8	2.2 ± 2.6	3.8 ± 2.6	*n* = 45, 16.30%	*n* = 15, 6.94%
Foot pain	6.0 ± 3.1	3.5 ± 3.6	2.3 ± 2.7	*n* = 70, 25.36%	*n* = 67, 31.02%
Hallux valgus	7.2 ± 2.7	4.2 ± 3.7	2.9 ± 3.0	*n* = 42, 15.22%	*n* = 43, 19.91%
Metatarsalgia	5.1 ± 2.9	1.6 ± 1.7	3.6 ± 2.9	*n* = 6, 2.17%	*n* = 1, 0.46%
Neuroma/bursa complex	6.9 ± 2.1	4.3 ± 3.4	2.7 ± 3.0	*n* = 18, 6.52%	*n* = 28, 12.96%
Osteoarthritis	7.0 ± 2.1	3.6 ± 3.4	3.4 ± 2.5	*n* = 35, 12.68%	*n* = 31, 14.35%
Plantar fasciitis	7.4 ± 1.8	2.8 ± 2.6	4.6 ± 2.8	*n* = 50, 18.12%	*n* = 21, 9.72%
*Total*	6.6 ± 2.7	3.4 ± 3.3	3.2 ± 2.8	*n* = 276, 100%	*n* = 216, 100%

In addition to the VAS pain scale, the patients' self‐reported percentage of condition improvement was also used (Table 5). All patients receiving care in the virtual MSK service had a significant improvement in the VAS pain scale at discharge, regardless of whether they were discharged to AHP services or self‐management. Patients who were referred to AHP services had a significantly higher initial VAS pain scale compared to those discharged on self‐management (*p* < 0.001). The most frequently reported groups for self‐reported percentage of condition improvement were 0%–24% and 75%–100%, with the former being reported by patients discharged to AHP services and the latter by those discharged to self‐management. There were no incident reports from the clinical or administrative team during the study, indicating that the service was safe and no serious occurred to patients and staff.

**TABLE 5:** Outcome measurements by discharge groups.

	At initial appointment	At discharge	VAS improvement (between an initial appointment and at discharge)	Significance
VAS pain scale (all)	6.6 ± 2.7	3.5 ± 3.3	3.2 ± 2.8	*p* < 0.0001
VAS pain scale (discharged to the self‐management group)	6.0 ± 2.7	1.3 ± 1.8	4.7 ± 2.6	*p* < 0.0001
VAS pain scale (discharged to the AHP group)	7.4 ± 2.4	6.2 ± 2.7	1.2 ± 1.7	*P* < 0.001
Self‐reported percentage of condition improvement (all)	N/A		N/A	N/A
0%–24%		*n* = 152, 30.9%		
25%–49%		*n* = 53, 10.8%		
50%–74%		*n* = 68, 13.8%		
75%–100%		*n* = 219, 44.5%		
Self‐reported percentage of condition improvement (discharged to the self‐management group)	N/A		N/A	N/A
0%–24%		*n* = 18, 6.5%		
25%–49%		*n* = 4, 1.5%		
50%–74%		*n* = 42, 15.2%		
75%–100%		*n* = 212, 76.8%		
Self‐reported percentage of condition improvement (discharged to the AHP group)	N/A		N/A	N/A
0%–24%		*n* = 134, 62.0%		
25%–49%		*n* = 49, 22.7%		
50%–74%		*n* = 26, 12.0%		
75%–100%		*n* = 7, 3.2%		

Abbreviation: N/A, not applicable.

## 4 DISCUSSION

The aim of this study was to evaluate the effectiveness of virtual MSK podiatry in providing timely intervention and management support to NHS patients. This evaluation primarily aimed to address the concern of extended waiting times and patient outcomes, as highlighted in previous literature [20]. Our service evaluated patient care at a time when access to healthcare was challenging due to the COVID‐19 pandemic.

A recent NHS England report from July 2023 shows that the average waiting time for primary healthcare services is currently 98.7 days [21]. The results from this service evaluation compare favourably as the waiting time between the initial referral and the first virtual consultation was 34.6 days. These findings highlight that the majority of patients received timely care by accepting a virtual consultation. By using virtual platforms, we were able to conduct remote assessments and provide guidance, minimising the need for face‐to‐face appointments and reducing waiting times.

As demonstrated in the results, the majority of patients reviewed in this study (56.5%) were discharged to self‐management with 76.8% of those patients reporting a 75%–100% improvement score (Table 5). National Health Service England encourage clinicians to promote self‐management and education to improve the patient's knowledge, skills and confidence in managing their own health. Self‐management reduces the NHS burden and has a positive impact upon health systems [21]. None of the patients experienced a deterioration in their symptoms as evidenced by the improved VAS scores (Table 5). Visual analogue scale score improvements for all patients were found to be statistically significant (*p* < 0.0001), with an average improvement of 3.2 ± 2.8. This result is comparable to a recent study investigating a group of 66 patients with Achilles Tendinopathy reported VAS score improvements of 3.0 and 3.4 for in‐person and virtual patients, respectively, when re‐assessed after 8 weeks from their baseline value [22]. In this work, a similarly modest VAS improvement of 3.6 ± 2.2 was also noted for the same pathology (Table 4). While our study investigated a large heterogenous group of patients, it is challenging to compare the results directly due to the differences in the studies' methods.

Furthermore, our service evaluation provides an indication into which were the better performing pathology categories that may be more suitable for virtual MSK services (Table 4). Although, patient numbers in each category are relatively low making it challenging to draw concrete conclusions, it can be noted that the categories of foot pain, hallux valgus and neuroma/bursa complex have not performed as well as other categories. Conversely, the categories of plantar fasciitis and flat foot appear to be better suited for virtual consultations due to their higher VAS improvements. This suggests that certain conditions may not be suitable for referral to virtual MSK services as a first‐line option. Future studies comparing face‐to‐face and virtual consultations are recommended to further explore this. Furthermore, future studies should consider a more extensive range of pathology categories than those employed in this study, which was limited due to the categories used by the commissioning system.

Our results demonstrated that the prescription of foot orthoses, with or without an exercise rehabilitation plan, has demonstrated statistically significant improvements in VAS scores (Table 3). This is in agreement with other evidence showing that foot orthoses are a valuable and effective treatment option in MSK podiatry [23, 24], either as a standalone intervention or in conjunction with other modalities such as education, exercise rehabilitation plans and footwear modifications [25]. It is worth noting that the aim of this study is not to evaluate the effectiveness of foot orthoses, and thus, we are unable to confirm or disregard the placebo effect that may also be a factor [26].

The innovative approach of virtual MSK services not only ensured uninterrupted access to care during challenging circumstances but also showed potential for optimising the allocation of NHS resources by efficiently managing patient caseloads [27]. Virtual platforms have demonstrated promising potential in improving accessibility for both patients and clinicians, allowing for convenient and flexible service delivery. This approach aligns with NHS Long Term Plan (2019 to 2029) for digital health which emphasises the importance of utilising technology to enhance patient care and improve healthcare outcomes [3, 28]. It is important to personalise virtual platforms for patients' specific needs such as preferences, age, internet accessibility and familiarity with electronic devices. Virtual consultations could be a recommended option for eligible patients, promoting faster rehabilitation and improve quality of life [29, 30]. Creating appropriate written guidelines is necessary to address legal barriers to implementing virtual consultations. Several countries have taken measures to eliminate obstacles and extend their services [31]. Such initiatives can significantly improve access to care and patient outcomes in the field of MSK Podiatry.

From the service provider's perspective, the implementation of virtual MSK podiatry services also provided clinicians with the opportunity to step out of their comfort zones, explore and develop new clinical approaches. As with any new service, there were initial challenges, and the approach to virtual consultations evolved over time. Clinicians faced a learning curve as they adapted to delivering patient care through virtual means. The management of the first few patients seen virtually differed from that of the later patients, as clinicians gained confidence and expanded their capabilities. Initially, clinicians focused more on active listening skills, motivational interviewing, shared decision‐making and guided functional assessments during telephone calls. Video calls expanded the assessment process, and clinicians were able to undertake a more thorough the MSK assessment. As a result, patients were encouraged to attend video calls and benefit from a more comprehensive clinical assessment process. Once the procedure matured, patients were instructed to position their electronic devices vertically against the wall in a systematic manner, enabling clinicians to perform visual weight‐bearing assessments. Through these assessments, the podiatrists could make observations related to foot positioning and develop a better understanding of the patient's symptomatology. This method facilitated discussions and the provision of advice to patients regarding their treatment planning including recommendations of footwear, exercise rehabilitation and orthotic prescription. As a result, the final assessment process was developed in an iterative manner with clinicians sharing experiences as their confidence grew to develop a mature system. Effective communication and teamwork have played a pivotal role in the development and improvements of both operational and clinical aspects of this service. These lessons learnt are valuable for future service provision, offering insights to other practices considering the adoption of similar procedures.

The establishment of guidelines through workshops and educational programs for fellow podiatrists can facilitate the dissemination of best practices and ensure the delivery of consistent high‐quality virtual MSK podiatry services. These guidelines should address important aspects such as patient selection criteria, appropriate use of virtual platforms, safety, data protection, health and wellbeing of the clinician, safeguarding adults and children, standards for clinical communication and record keeping. National podiatric specific best practice guidelines require development to support podiatrists delivering digital services. By establishing comprehensive guidelines, virtual services can be widely accepted and fully integrated into healthcare systems, providing improved access to care and enhancing the patient experience.

Due to the nature of the commissioned virtual MSK service, we did not offer any face‐to‐face consultations and are unable to provide any comparison of outcomes between the virtual and face‐to‐face MSK services. Thus, the focus was on the virtual MSK service alone. Also, at the point of triage, patients did not have a choice whether they would be accessing virtual or face‐to‐face MSK services. As evidenced by 82 non‐engagements (14.3%) with 24 patients explicitly stating that they did not wish to access virtual MSK service, future services should consider whether providing patients with a choice may be a better option for improved engagement with virtual MSK services. In addition, it is important to acknowledge that the study was conducted during the COVID‐19 pandemic. The impact of this unique period on our findings may not fully reflect the complete potential of virtual MSK podiatry services in a post‐pandemic setting [32]. Furthermore, the data were collected via phone and virtual consultations in a non‐anonymous manner which may be susceptible to potential bias.

Although the introduction of the virtual MSK service was initially in response to the pandemic, it quickly became apparent that it presents an opportunity for integrating such services into routine clinical practice. To fully realise the potential of these services, future investigations should explore their long‐term impact beyond the pandemic era. Additionally, future studies could investigate larger and more diverse sample sizes or focused investigations of specific pathology categories. These investigations could hold the potential of shaping the development of best practice guidelines, tailored specifically to MSK podiatry services, ensuring the optimisation of virtual consultations and the continuous enhancement of the quality of care provided to patients. Through the ongoing evaluation and refinement of these services, podiatrists can effectively adapt to patients' needs and utilise the advantages of technological advancements.

## 5 CONCLUSIONS

This study provided evidence that the delivery of a virtual MSK podiatry service was statistically significant in reducing patients' pain and provides evidence supporting the efficacy of a digital service delivering conservative management plans to NHS patients. The virtual MSK service provided shorter waiting times between a referral acceptance and an initial consultation which directly benefitted patients and helped to reduce NHS waiting lists. Integrating virtual services to existing traditional face‐to‐face practices aligns well with The NHS Long Term Plan for digital health. The use of virtual platforms also offers convenience and accessibility for patients with favourable treatment outcomes. In response to the development of the virtual MSK service, internal guidelines were created to suit the needs of the podiatry practice in delivering virtual consultations. Future virtual services, particularly MSK podiatry, will benefit from clear best practice guidelines to ensure high quality of service delivery while maintaining the required governance. Virtual podiatry services can revolutionise healthcare delivery by offering safe, effective and convenient treatment options for patients and podiatrists. The economic, environmental and societal benefits of virtual consultations highlight the importance of further research to ensure their widespread acceptance, safe and efficient utilisation in clinical practice.

## AUTHOR CONTRIBUTIONS

**Agalliu Brunilda**: Conceptualization; data curation; formal analysis; funding acquisition; investigation; methodology; project administration; resources; supervision; software; validation; visualization; writing – original draft; writing – review & editing. **Tunprasert Thanaporn**: Software; writing – review & editing. **Walton Tom**: Resources; supervision; writing – review & editing. **McManus Liam**: Project administration; resources; supervision; writing – review & editing.

## CONFLICT OF INTEREST STATEMENT

Statement and Declarations: BA, TW and LM acknowledge funding from Algeos Limited medical supply group UK. TT has no conflicts of interest to declare.

## ETHICS STATEMENT

This study is a retrospective evaluation of a virtual musculoskeletal (MSK) podiatry service implemented for NHS patients by a private podiatry clinic. The evaluation analyses existing data and does not involve collecting new data from participants. We ensured patient privacy and data protection by obtaining approval from both the NHS Trust and the private podiatry clinic, acting as the data controllers. This ensured informed consent practices were followed according to NHS regulations. After consultation with the NHS Research and Development team, it was determined that this retrospective evaluation did not require a formal ethical approval from a NHS Research Ethics Committee. We are committed to contributing research with respect for patient privacy and following relevant data protection regulations.

## Data Availability

Research data are not shared.
